# SELF-EFFICACY AND TEAM LEADER EQUITY MATTER: A STUDY OF ACTIVE AGING AT WORK

**DOI:** 10.1093/geroni/igz038.2808

**Published:** 2019-11-08

**Authors:** Mi Sun Choi, Holly Dabelko-Schoeny, Mo Yee Lee, Alicia Bunger

**Affiliations:** The Ohio State University, Columbus, Ohio, United States

## Abstract

Many older Americans have decided to remain in the labor market beyond the traditional retirement age, suggesting the need for companies to consider human resource initiatives to retain and support the aging workforce. Applying active aging concepts, which emphasize older adults’ active roles through participation in social and economic activities for healthy later life, to the workplace could be helpful for developing programs that enhance the health, well-being, and work outcomes of older workers. Despite the expected benefits of active aging at work for older workers’ overall well-being, little research has been conducted on what personal and team factors impact on outcomes of active aging at work; what mechanism exists in the links between factors and outcomes in the contemporary workplace. The current study tested the validity of an active aging framework using the Age and Generations Study data. We analyzed responses of 508 American workers aged 50 and older using structural equation modeling. Results showed that perceived self-efficacy was a strong predictor of engagement, mental health, and performance, whereas perceived leader equity predicted only engagement. Also, work engagement was powerful mechanism for promoting older adults’ mental health; engagement mediated the relationship between perceived self-efficacy and the relationship between leader equity and mental health. The findings highlight how important it is for employers to invest in human capital, suggesting human resource programs should focus on strategies that target older adults’ engagement through tailored self-efficacy programs and diversity leadership training programs. Such attempts would contribute to the well-being of older workers.

